# The Emerging Role of ATP-Dependent Chromatin Remodeling in Memory and Substance Use Disorders

**DOI:** 10.3390/ijms21186816

**Published:** 2020-09-17

**Authors:** Alberto J. López, Julia K. Hecking, André O. White

**Affiliations:** 1 Department of Pharmacology, Vanderbilt University School of Medicine, Nashville, TN 37232, USA; alberto.lopez@vanderbilt.edu; 2 Department of Biological Sciences, Mount Holyoke College, South Hadley, MA 01075, USA; hecki22j@mtholyoke.edu

**Keywords:** epigenetics, long-term memory, addiction, nucleosome remodeling, plasticity, neurodevelopment, substance use disorder

## Abstract

Long-term memory formation requires coordinated regulation of gene expression and persistent changes in cell function. For decades, research has implicated histone modifications in regulating chromatin compaction necessary for experience-dependent changes to gene expression and cell function during memory formation. Recent evidence suggests that another epigenetic mechanism, ATP-dependent chromatin remodeling, works in concert with the histone-modifying enzymes to produce large-scale changes to chromatin structure. This review examines how histone-modifying enzymes and chromatin remodelers restructure chromatin to facilitate memory formation. We highlight the emerging evidence implicating ATP-dependent chromatin remodeling as an essential mechanism that mediates activity-dependent gene expression, plasticity, and cell function in developing and adult brains. Finally, we discuss how studies that target chromatin remodelers have expanded our understanding of the role that these complexes play in substance use disorders.

## 1. Introduction

Recent research has advanced our understanding of the molecular infrastructure that facilitates the formation of long-term memories. Long-term memory formation requires experience-dependent changes in gene expression and neuronal function [1]. In the nucleus, gene accessibility is largely influenced by the structure of chromatin. Generally, 147 base pairs of genomic DNA are spooled around a histone octamer to form functional chromatin [2]. This chromatin is highly compacted in the nucleus, therefore enzymes that control chromatin structure and subsequent access to DNA, play an integral role in regulating gene expression. Interactions between DNA and the core histone proteins are routinely adjusted through epigenetic mechanisms such as DNA methylation, histone modification, histone variant insertion, and chromatin remodeling [3]. Even though epigenetic mechanisms do not change the DNA sequence, they can confer persistent cell function changes that last beyond the life of an individual protein [4]. Understanding the role of epigenetics in these long-term cell function changes may provide insight into how persistent memories are maintained in normal and disease states.

Maladaptive circuit plasticity contributes substantially to the enduring nature of some behavioral disorders, including major depressive disorder (MDD) and substance use disorder (SUD). For example, in substance use disorder (SUD), drug-associated memories often drive drug-seeking behaviors and an increased risk of relapse after the cessation of drug use [5,6]. Over the last four decades, advancements in memory research have established epigenetic mechanisms––such as DNA methylation and histone modification––as being integral to the formation and maintenance of these resilient drug-associated memories [7]. However, recent data have broadened our understanding of the role of chromatin remodeling in MDD and SUDs. In this review, we highlight prominent examples of histone modifications in canonical long-term memory formation and explore how this seminal work provides a framework for understanding the role of other epigenetic mechanisms in the development and reinforcement of SUDs. We discuss chromatin remodeling as an understudied yet robust epigenetic mechanism underlying transcription, cell function, and cognition in the developing and adult brain. Lastly, we consider how chromatin remodeling complexes (CRCs) may interact with the well-characterized library of histone modifications to induce large-scale changes to chromatin structure and synaptic function in SUDs.

## 2. Epigenetics in Learning and Memory

### 2.1. Overview

Epigenetic mechanisms regulate chromatin compaction in a manner that establishes an environment that is either permissive or obstructive to gene expression during memory formation. In general, epigenetic mechanisms regulate chromatin in three ways: (1) nucleotide modifications (DNA and RNA); (2) histone modifications, whether at amino-terminal tails or through histone variant exchange; and (3) chromatin remodeling by ATP-dependent chromatin remodeling complexes [3]. The roles of histone modifications and DNA methylation in learning and memory have been reviewed considerably [4,8,9,10,11,12,13]. Below, we focus on histone modification’s role in memory, as their lysine targets can serve as recognition sites for ATP-dependent chromatin remodelers. This review focuses on how epigenetic mechanisms may directly communicate with each other to regulate chromatin structure and gene expression. Therefore, this focus necessarily excludes non-coding RNAs, which can indirectly regulate chromatin structure as well as directly target RNA after transcription has occurred [14,15]. While a promising avenue for future research, the mechanisms by which non-coding RNAs fulfill their role in memory are poorly understood. It should also be noted that histone variants can be incorporated in place of core histone proteins by ATP-dependent chromatin remodeling complexes, thereby altering chromatin compaction [16]. Nascent evidence suggests that histone variant exchange may regulate local transcription necessary for synaptic plasticity and memory formation, however, this research is ongoing.

### 2.2. Histone Modifications in Learning and Memory

Post-translational modifications are critical regulators of chromatin compaction. In the field of learning and memory, the best-studied post-translational modification is histone acetylation, a process which is bi-directionally regulated by histone acetyltransferases (HATs) and histone deacetylases (HDACs). Briefly, HAT and HDAC enzymes compete for influence over histone-DNA contacts through the addition and removal of acetyl groups, respectively. Lysine residues within the amino-terminal tails of histones form covalent bonds with the negatively charged DNA phosphate backbone. Histone acetylation adds acetyl groups to these lysine residues, thereby negating their positive charge and breaking their bond with DNA—leaving DNA in a less compact state. Generally, HAT activity is considered permissive to gene expression, whereas HDAC activity is restrictive. Evidence implicating histone acetylation in learning and memory processes came as early as 1979 [17]. Using labeled-acetate, Schmitt and Matthies demonstrated that, following fear conditioning, acetyl groups were added to histones in the rat hippocampus. As this initial finding, mounting evidence has shown that histone acetylation is required for activity-dependent gene expression in long-term memory processes.

In the hippocampus, histone acetylation has been shown to regulate activity-dependent gene expression during memory processes. Researchers have proposed the molecular brake pad hypothesis to explain how the histone acetylation state regulates these memory processes. The molecular brake pad hypothesis posits that under basal conditions, gene expression that is necessary for memory consolidation is inhibited by HDACs and their associated co-repressors (the molecular brake pad). In this model, a strong stimulus promotes second messengers (e.g., leads to enhanced calcium influx) binding to HDACs and their corepressors, releasing them from the promotors of memory-related genes. Thereafter, histone acetylation, by HATs, enhance the recruitment of coactivators to promote the expression of memory-linked genes [18]. Consistent with this view, hippocampal studies have demonstrated that the inhibition of HDAC activity is necessary and sufficient for long-term memory formation in novel object location [19,20], contextual fear conditioning [21,22,23], and coincides with increases histone acetylation. Remote memories can be resistant to memory updating during reconsolidation. However, inhibition of HDAC2 function in the hippocampus results in histone hyperacetylation while enabling the modification of remote memories [24].

Over the last decade, numerous studies targeting histone-modifying enzymes have generally supported the aforementioned role of HDAC and HATs in memory. Systemic administration of HDAC inhibitors sodium butyrate and trichostatin A enhance contextual fear memory [19,25] and fear extinction [26,27], whereas homozygous deletion of HDAC2 enhances both cue and contextual fear memory [28]. With regards to reward learning, systemic administration of the HDAC inhibitor RGFP966 leads to lysine-specific (i.e., H4K8 and H3K14) enhancements in acetylation while facilitating the extinction of cocaine-induced conditioned place preference [29]. RGFP966 strengthens plasticity and memory specificity in the auditory cortex [30]. Conversely, inhibiting the function of HATs (e.g., CBP) has the opposite effect of HDAC inhibition, leading to aberrant gene expression profiles, deficits in synaptic plasticity, and impaired memory formation [23,31,32]. Together, these findings and numerous others have established HATs and HDACs as potent regulators of transcription necessary for memory processes.

While histone acetylation is a canonically permissive mark, the role of histone methylation in gene regulation during memory formation remains opaque. Added by histone methyltransferases (HMTs), methyl groups on histone lysine residues can occur in mono-, di-, and tri-forms with each methylation variant capable of exerting unique influence over gene accessibility. The methylation status of histones is also tightly regulated by lysine-specific demethylases (KDMs), which remove methyl groups. HMTs, including Mll, KMT2B, and G9a are required for memory formation, as their deletion blocks activity-dependent gene expression and lead deficits in long-term memory formation [33,34]. Furthermore, the loss of HMT function has been demonstrated to impair the amygdala function, as G9a deletion in the lateral amygdala impairs fear memory [35].

The role of KDMs in memory formation remains less clear. Inhibiting histone demethylation by blocking KDM1a enhances fear conditioning memory [35]. Whereas, KDM1A loss-of-function and *Phf8* genetic deletion have been demonstrated to induce deficits in long-term memory formation and learning in the hippocampus and amygdala [36,37,38]. These non-linear results emphasize the complex role of histone methylation in regulating gene expression; some histone methylation marks have been shown to be critical for gene activation (such as H3K4, H3K48, and H3K79), whereas, other methylation marks are thought to be essential for gene inactivation (such as H3K9 and H3K27; see Table 1). Further studies are crucial to elucidate the mechanisms by which methylation modifying enzymes alter cognitive function.

While histone acetylation and methylation are the most well-studied histone modifications, several other histone modifications have potential roles in cognition. For example, poly-ADP ribosylation (PARylation) is a unique histone mark that accumulates in response to DNA damage and cellular activity [39]. Furthermore, poly-ADP-ribose polymerase 1 (PARP-1), one of the primary enzymes responsible for histone PARylation, is required for activity-dependent gene expression in neurons [40]. PARylation via PARP-1 is necessary for transcription-dependent long-term memory formation in the hippocampus [41]. Research suggests that PARylation evicts linker histone H1 from the nucleosome and is essential for subsequent changes in nucleosome conformation [40]. However, the exact mechanism by which PARylation alters transcription or nucleosome conformation remains unclear, it is an area of research that requires more exploration.

Histone modifications can combine on a single histone to modify gene expression [14]. Histone H3 phosphorylation accumulates in response to neuronal activity [42,43] and supports long-term memory formation [44]. One mechanism by which phosphorylation confers epigenetic permissiveness is through the subsequent recruitment of other histone-modifying enzymes. Specifically, phosphorylation of H3S10 is known to increase the binding of histone acetyltransferase KAT2A/GCN5 to H3K9 and H3K14 residues [45]. KAT2A activity and subsequent acetylation have been linked to hippocampal LTP and memory formation [46]. Therefore, it is likely that phosphorylation may act as a scaffold by facilitating the addition of other histone modifications.

Histone acetylation has also been shown to interact with other epigenetic mechanisms (i.e., histone methylation and chromatin remodeling) to regulate DNA access. At least one study implicates methylation in priming genes for future activation by HATs. Wang et al. (2010) showed that after HDAC inhibition, subsequent histone acetylation at target genes required H3K4 methylation [47]. It is also widely accepted that bromodomain-containing chromatin remodelers read histones’ acetylation state to form complexes that either promote or silence gene expression. Overall, this view of histone modification serving as an interface between various epigenetic mechanisms adds further complexity to our understanding of epigenetics’ role in memory.

## 3. Introduction to Chromatin Remodeling Complexes

ATP-dependent chromatin remodelers have established roles in regulating gene expression. Chromatin remodeling complexes (CRCs) are made up of multiple specialized proteins that play essential roles in packaging and regulating access to the genome throughout the cell cycle. CRCs are currently organized into four families: (1) SWI/SNF (switching defective/sucrose nonfermenting), (2) ISWI (imitation switch), (3) CHD (chromodomain, helicase, DNA binding), and (4) INO80 (inositol requiring 80) [63]. These families share features including an affinity for nucleosomes; specialized protein subunits or domains (for interacting with histone modifications, transcription factors, and chromatin); and DNA-dependent ATPase activity [63,64]. In concert with other epigenetic and non-epigenetic factors, CRCs facilitate dynamic processes that include DNA replication, DNA repair, DNA recombination, and transcription. The effect of CRC activity on gene expression is primarily determined by their unique ATPase catalytic domains and the binding domains of the associated subunits [63]. Each CRC employs their respective ATPase subunit to hydrolyze ATP, resulting in increased nucleosome mobility by disrupting contacts between nucleosomes and DNA [65]. As such, individual CRC families are often discussed concerning their dedicated DNA-dependent ATPase. For example, the mammalian analog of the yeast SWI/SNF complex utilizes BRG or hBRM ATPases and is therefore named BAF (BRG or hBRM Associated Factors). In contrast, ISWI complexes employ SMARCA1/5 ATP-ase containing proteins. Below, we discuss the current state of knowledge about the role of CRCs in cognition. We highlight the BAF and ISWI families as they have been the most thoroughly explored.

## 4. Chromatin Remodeling Complexes in Neurodevelopment

### 4.1. ISWI

Although expressed throughout organismal development, both BAF and ISWI complexes show evidence for unique neuron-specific functions. The family of ISWI proteins are characterized by a conserved SANT-domain that allows for interaction with histone modifications and targeted ATPase dependent remodeling (see Table 2). SMARCA1 (SNF2L) has been identified as the mammalian homolog to the drosophila ISW1 ATPase, whereas SMARCA5 (SNF2H) has been identified as the homolog to the ISW2 ATPase. It has been demonstrated that these core ATPases alone can induce nucleosome remodeling, independent of other ISWI-complex subunits. Though SMARCA5 is expressed in most cell-types throughout development, SMARCA1 is predominantly expressed in post-mitotic neurons, suggesting a role for SMARCA1 in neuron-specific gene regulation [66]. This ability to independently interact with nucleosomes and their modifications, suggests ISWI and its homologs are critical gene regulators throughout development. In support of this view, loss of ISWI function leads to the misregulation of *Pax6* and *Shh*, critical genes in neuronal development [67,68]. These deficits can be rescued with overexpression of functional ISWI, which suggests that normal ISWI function is necessary and sufficient for the proper regulation of these neurodevelopmental genes.

Both SMARCA1 and SMARCA5 are necessary to maintain proper mammalian nervous system development. For example, SMARCA5 knockouts cause downregulated *Engrailed-1* expression leading to severe cognitive and motor deficits due to disrupted histone H1/chromatin interactions at the *Engrailed-1* locus during Purkinje and granule cerebellar development [69,70]. SMARCA5-dependent regulation of *Engrailed-1* can be rescued through temporally specific SMARCA1 compensation; however, loss of both SMARCA1/5 is post-natal lethal. In contrast, SMARCA1 is also able to remodel chromatin for targeted downregulation of genes throughout neuronal development. SMARCA1 mutants exhibit aberrant upregulation of *Foxg1*, resulting in excess neural proliferation. Moreover, SMARCA1/5 regulates neuronal cell fate; loss of SMARCA1 delays the development of dopaminergic cell identity and maturation, whereas SMARCA1 gain of function induces dopaminergic differentiation. The ISWI family of nucleosome remodelers are vital enzymes to neuronal development and differentiation.

Due to its role in neuronal proliferation and differentiation, SMARCA1 has been identified as a candidate gene for neurogenetic disorders. Genetic loss of function in SMARCA1 was identified in families with individuals displaying neurodevelopmental and facial dysmorphism similar to Coffin-Siris syndrome [71]. Single nucleotide polymorphisms (SNPs) studies have identified missense mutations within *SMARCA1* in schizoaffective families [72]. As an evolutionarily conserved family of nucleosome remodelers, ISWIs such as SMARCA1/5 may have roles in gene expression regulation necessary for cognition that is dissociable from their role in gene expression regulation throughout development; however, the role of the ISWI family in adult cognition requires more exploration.

### 4.2. nBAF

The role of BAF complexes in neurodevelopment has been thoroughly studied in healthy neurodevelopment and BAF-linked neurodevelopmental disorders. BAF complexes are comprised of 15–20 accessory protein subunits that can be exchanged for the complexes to fulfill their diverse roles in cell fate decision and cell function (see Table 2). While the core subunits’ activity can maintain BAF ATPase function, the combinatorial assembly of other BAF-specific subunits around these core enzymes can alter the overall complex affinities and functions. In neuroscience, neuron-specific BAF (nBAF) and its dedicated subunits (BAF53b, BAF45b/c, and CREST) have been the most examined BAF complex. The integration of these dedicated subunits into the BAF complex enables targeted regulation of genes required for proper brain development. For example, preventing the integration of BAF53b or BAF45b/c into the BAF complex during development prevents the transition from neuronal progenitor cells to post-mitotic neurons [73,74]. Knockout studies have shown that loss of BAF53b leads to aberrant activity-dependent dendritic development [73,75,76,77]. These neuron-specific subunits guide activity-dependent chromatin remodelers to genes necessary for axonal and dendritic arborization (such as *Ephexin1*), providing epigenetic regulation of activity-regulated genes throughout development [73].

In addition to these cellular deficits linked to nBAF loss of function, BAF subunits have been implicated in neurodegenerative diseases and neurodevelopmental disorders (NDDs). Human studies suggest that autism spectrum disorder (ASD), intellectual disability (ID), Coffin-Siris syndrome (CSS), and Nicolaides–Baraitser syndrome (NBS), may have roots in mutations in BAF complex subunits [78]. The clearest evidence for mutations of BAF subunits causing an NDD comes from a recent study from Wenderski and colleagues (2020) [79]. They performed whole-exome sequencing on human patients with recessive autism to identify genes that may significantly contribute to the disorder. This study compared a cohort of 135 patients with recessive ASD to a cohort of 256 patients with non-ASD recessive NDD. They found that BAF53b (also known as Actl6b) was significantly mutated in the recessive ASD cohort but not in the non-ASD NDD cohort. Additionally, the researchers further identified six families from the recessive ASD cohort that had homozygous variants of BAF53b. In each case, individuals with homozygous variants of BAF53b displayed an ASD phenotype, whereas individuals with heterozygous BAF53b mutations did not.

Based on previous research, the researchers predicted that BAF53b played an integral role in the nBAF complex’s composition. This prediction was supported when the expression of the BAF53b missense mutant protein in HEK293T cells, human embryonic stem cells, and BAF53b^−/−^ primary mouse neurons resulted in the mutant protein almost always failing to form a complex with other nBAF subunits [79]. This work is one of the first to address how specific mutations may affect the structure and activity of a CRC to give rise to cognitive disorders. Together, research targeting BAF CRCs demonstrates the integral role of BAF subunits in neurodevelopment, cell function, and cognition. However, it remains unclear whether the role BAF complexes play in adult cognition arises solely from their role in neurodevelopment, or whether their activity in post-mitotic neurons regulates gene expression mediating adult cognition.

**Table 2:** Chromatin remodeling complexes regulate long-term memory.

Remodeling Complex	Neuron-Relevant Subunit	Target Residues	Transcriptional Effect	References
nBAF	BAF53B/ACTL6B CREST BAF45B BAF45C	Acetylated Histones (via SMARCA bromodomains)	Permissive (Mor1, Bdnf, Mef2d, Cap2, Dbn1) Repressive (Fos, Fosl2, Fosb, Junb)	[78,79,80,81,82,83]
ISWI	SMARCA1 SMARCA5 BAZ1A BAZ1B BAZ2B CECR2 RSF1	Acetylated Histones (via CECR2 and SMARCA SANT-domains; BAZ bromodomains) Methylated Histones (via chromodomains and PHDs)	Permissive	[84,85,86,87,88,89,90,91]
NuRD	Mi-2a/b MBD3 MTA1-3 RbAp46 RbAp48 HDAC1/2	Histone Lysine Residue (via HDAC1/2 bromodomains) Methylated Histones (via Mi-2a/b chromodomains)	Repressive	[92,93,94,95,96,97,98,99,100,101,102,103,104]

## 5. Chromatin Remodeling Complexes in Adult Cognitive Function

### 5.1. ISWI Subunits Regulate Depressive-Like Phenotypes

The BAF family of CRCs remains the most studied in relation to adult cognition. However, the ISWI family contains several components that make them ideal candidates to examine the role of ATP-dependent chromatin remodeling in cognition. The ISWI family of remodelers (reviewed in [97]) includes dNURF, dCHRAC, dACF, and hNoRC complexes. These complexes contain an ATPase domain and a HAND-SANT-SLIDE domain that supports binding to DNA, nucleosomes, and potentially influences the directionality of nucleosome movement (reviewed more extensively [63,98]). Additionally, accessory proteins provide further DNA and histone binding specificity to promote changes to nucleosome spacing and transcription regulation. Through screens of cDNA libraries and databases, Jones et al. (1999) identified four genes (BAZ1A, BAZ1B, BAZ2A, and BAZ2B) that contain bromodomains that encode for accessory proteins that associate with the ISWI ATPase to form the ACF (ATP-utilizing chromatin assembly and remodeling factor) chromatin remodeler complex [99]. The ability of proteins with bromodomains to regulate transcription through histone acetylation has been thoroughly examined in memory formation and substance use disorder [100,101,102,103], however, it is unclear to what extent ISWI complexes’ regulation of gene expression contribute to cognition.

There is support for a role for ISWI remodeling subunits in depressive-like behaviors. Sun et al. (2007) used a mouse model of depression to examine mRNA expression profile in subunits from the four families of chromatin remodeling complexes (SWI/SNF, ISWI, CHD, and INO80) in the nucleus accumbens (NAc) [104]. They identified increases in BAZ1A mRNA and protein correlated with depression in humans and depression-related behaviors in mice. Though both BAZ1A and the closely related BAZ1B, were shown to associate with SMARCA5 to form ACF complexes, BAZ1A-SMARCA5, complexes appeared to increase in the NAc after chronic social defeat stress, while BAZ1B-SMARCA5 complexes did not. BAZ1A but not BAZ1B or SMARCA5 increases were observed in the NAc of postmortem brains from depressed humans. Together, these findings suggest that changes induced by depression-like models are specifically associated with the ACF complex’s BAZ1A accessory subunit.

These results left open the possibility that BAZ1A was not directly driving the depression susceptibility phenotype. To address this, the authors explored whether overexpression of the BAZ1A-SMARCA5 composition of the ACF complex could transform a subthreshold social defeat training into one that produced increased susceptibility [104]. This type of study follows an increasing body of evidence that suggests that during a weak learning event, repressive epigenetic mechanisms prevent transcription necessary for long-term memory formation: thereby, setting a threshold of cell activation that must be exceeded for memory formation [18]. Manipulations that remove these corepressors (or increase coactivator function) appear to lower the cell activation required for memory formation by enhancing the expression of memory-related genes. Sun et al. further demonstrates mice with a viral-mediated overexpression of BAZ1A-SMARCA displayed a depression-like phenotype after subthreshold social defeat training. Given that the overexpression of BAZ1A and SMARCA5 together but not individually results in a susceptibility phenotype after subthreshold training, it is possible that the BAZ1A-SMARCA complex is instrumental in regulating the formation of depression-like behaviors. Importantly, this work represents one of the first studies to suggest a role for nucleosome remodeling within the framework of the molecular brake pad hypothesis.

### 5.2. nBAF is Essential for Synaptic Plasticity and Memory Processes

Converging evidence now supports a role for nBAF in consolidating long-term memories in the adult brain. To date, one of the neuron-specific subunits of the nBAF complex, BAF53B, has been targeted in the hippocampus and amygdala to assess its role in memory formation [80,105]. Mutant mice with heterozygous knockout (BAF53b^+/−^) or dominant-negative mutation of BAF53b (BAF53bΔHD) display impaired object location memory and contextual fear memory, both hippocampus-dependent tasks. Given the aforementioned role of BAF53b in neurodevelopment, it was an open question as to whether deficits were related to memory processes in the adult or aberrant neuronal differentiation that occurred during development. To assess the role of BAF53b in the adult brain, Vogel-Ciernia and colleagues reintroduced adeno-associated virus expressing wildtype *Baf53b* into the dorsal hippocampus of mutant mice before training [82]. Viral overexpression of *Baf53b* in the dorsal hippocampus successfully rescued deficits in these hippocampus-dependent memory tasks.

The role of BAF53b in memory is supported by subsequent experiments showing that BAF53b expression in the amygdala is required for cue fear conditioning [105]. Knockdown of endogenous BAF53b expression via shRNA, in the lateral amygdala, impairs cue fear memory formation. Conversely, BAF53b overexpression using a herpes simplex virus-based vector enhances cue fear memory formation. This memory enhancement was persistent, affecting both recent (1-day post-training) and remote (29 days post-training) cue fear memory. The persistence of BAF53b-related memory enhancement is reminiscent of the enhancements observed after HDAC inhibition (reviewed in [100]). It is important to note that in each of the experiments discussed above, animals with BAF53b manipulations displayed normal short-term memory: an indication that they were able to perform the task. It appears that their deficits were a failure to consolidate long-term memories.

While assessing the role of BAF53b in memory, researchers also found evidence that BAF53b is required for normal synaptic function in the adult brain. Long-term potentiation (LTP) has long been considered a transcription-dependent synaptic mechanism underlying memory processes. Hippocampal slices from BAF53b^+/−^ mutant mice show a failure to maintain LTP and an absence of phosphorylated cofilin (p-cofilin) induction [83]. Given that BAF53b is required for the localization of nBAF to the promoter of target genes during activity-dependent synapse development [73], it is unsurprising that that BAF53b mutant mice display impairments in synaptic function. These findings raised the question of whether BAF53b-related deficits in synaptic function are a permanent feature of an aberrantly developed brain.

In the following study, Vogel-Ciernia and colleagues showed that subdomain 2 of BAF53b is critical for BAF53b’s ability to regulate synaptic function and memory formation in the adult brain [82,83]. Previous research identified BAF53b’s subdomain 2 (amino acids 39–82) as showing the least similarity to its non-neuronal homolog, BAF53a [106,107,108]. In BAF53b^−/−^ neuronal cultures, substituting BAF53a’s subdomain 2 with the subdomain 2 of BAF53b was sufficient to rescue deficits in dendritic outgrowth and gene expression [73]. By contrast, BAF53b with the subdomain 2 from BAF53a in place of its own, was incapable of rescuing the deficits mentioned above in gene expression and dendritic outgrowth. Therefore, to better understand how BAF53b may be regulating synaptic function, Vogel-Ciernia and colleagues generated mice that overexpressed a mutant form of BAF53b, which lacked the subdomain 2 (BAF53bΔSB2) [83]. BAF53bΔSB2 mice displayed the predicted impairments in LTP and memory, along with concomitant reductions in p-cofilin phosphorylation. In BAF53bΔSB2 mice, deficits in hippocampal synaptic plasticity and hippocampus-dependent memory were mitigated by overexpressing a phosphorylation mimetic of cofilin in the dorsal hippocampus. Overall, these findings suggest that nBAF, through the subdomain 2 of BAF53b, regulate gene expression, and synaptic plasticity in a manner that supports long-memory formation. These deficits are not permanent which suggests that nBAF may have a role in memory formation that is independent of its role in neurodevelopment.

**Figure 1:**
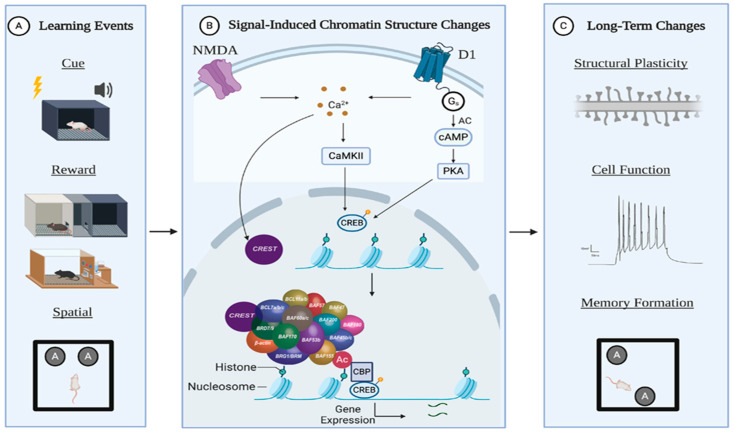
Model for recruitment of nBAF complex and histone acetylases in plasticity-associated events. Long-term memory formation learning about environment for optimizing future behaviors. This has been primarily modeled in rodents through (**A**) cue associations, reward-associated behaviors, and general spatial learning. These various training paradigms converge in their ability to alter neuronal signaling in specific hubs of the neural circuitry regulating reward and long-term memory, most commonly through altered dopaminergic and glutamatergic signaling. (**B**, top). Both dopaminergic (through D1-receptors) and glutamatergic (through NMDA receptors) signaling alter internal Ca^2+^ levels, converging on calcium signaling cascades in the nucleus. Ultimately, various epigenetic modifying enzymes respond to increased nuclear calcium to alter chromatin structure. Specifically, the neuron-specific subunit, CREST, responds to calcium, interacts with other calcium-dependent modifiers (such as CBP), and engages the nBAF-dependent nucleosome remodeling modification required for activity-dependent gene expression [77,110]. The output of this complex series of molecular signaling events is long-lasting changes to (**C**) synaptic plasticity, neural circuit function, and long-term memory formation. Drugs of abuse (such as cocaine) engage these molecular pathways to ultimately remodel the neural circuitry for sustained drug-associated memories and drug-seeking behaviors.

Although the BAF53b subunit has been thoroughly studied in regard to nBAF function, the CREST subunit is understudied. As with BAF53b, CREST is a neuron-specific subunit that plays a substantial role during development. Loss of CREST leads to aberrant activity-dependent dendritic development [73,75,77], and CREST mutations have recently been identified in ALS patients [77,109]. As CREST is a calcium-sensitive subunit, a potential mechanism for activity-dependent nBAF chromatin remodeling includes CREST responding to changes in nuclear calcium caused by a learning event (Figure 1). Moreover, CREST is a known binding partner of CBP, a histone acetyltransferase, implicating interactive functions between histone acetylation and chromatin remodeling in activity-dependent gene expression, learning, and memory [75]. Further studies should explore the CREST-dependent nBAF activation and subunit-specific targeting of nBAF function during memory processes.

## 6. Epigenetics in Substance Use Disorders

### 6.1. Histone Modifications

Over the past several decades, there has been increasing pre-clinical data characterizing SUD as a disease of aberrant learning and memory formation [4,111,112,113,114]. During SUD, aberrant learning manifests in strengthened associative processes with drug- and reward-related cues, among others [115,116]. Both normal learning and the aberrant learning seen in SUD share common neural circuitry and underlying cellular mechanisms to ultimately drive behavioral outcomes [111,117,118,119,120,121]. There is a growing appreciation that the molecular mechanisms that are critical for synaptic plasticity and memory formation are also hijacked by drugs of abuse to support drug-seeking behaviors and the risk of relapse. As such, much of the pioneering work examining histone-modifying enzymes in learning and memory have been successfully applied to SUDs.

Drugs of abuse are known to induce widespread molecular adaptions throughout the nervous system, including long-lasting modifications to chromatin accessibility and gene expression through the recruitment of various histone-modifying enzymes, such as HATs, HDACs, and KMTs (for a full review of histone modifications in cocaine-associated behaviors see López et al. 2020 [9]). For example, in response to acute cocaine, D1-MSNs show a significant upregulation of several permissive marks, such as H3K4me3, pH3S10, H3K14ac, and H4K5ac. Conversely, D2-MSNs show robust induction of various repressive marks, including H3K9 methylation, in response to cocaine exposure [29,122,123,124,125]. These cocaine-induced changes in acetylation strongly implicate HDAC and HAT enzymes in cocaine-associated behaviors. Indeed, disengagement of Class I HDACs significantly alters behavioral strategies used in cocaine self-administration [126,127,128,129].

In addition to altered acetylation mechanisms, histone methylation regulatory enzymes are also linked to cocaine response and cocaine-associated behaviors. Specifically, Kmt4, Kdm5a, -6a, -6b, and -7a are all increased in the prefrontal cortex following cocaine self-administration, whereas KDM6B is also increased in the PFC following cocaine withdrawal. Moreover, G9a is a critical regulator of cocaine motivation, self-administration, and cocaine-associated memory formation [130,131,132]. As histone modifications appear to underlie dysregulated learning in SUD, the field of neuroepigenetics could benefit from an examination of how other epigenetic mechanisms regulate the molecular, cellular, and circuit-level changes that occur in SUD.

### 6.2. ISWI and nBAF Chromatin Remodeling Complexes

As with learning and memory, mounting evidence suggests that CRCs are critical regulators of drug-induced conformational changes throughout the reward circuitry. For example, large-scale chromatin accessibility changes induced by cocaine have been identified in the PFC of rats using ATAC-seq [133]. In addition, cocaine-induced changes to active gene loci were found to be further enhanced by adolescent pre-exposure to the synthetic cannabinoid WIN 55,212-2 [133]. Although histone modifications confer changes in gene accessibility, these large-scale changes identified using ATAC-seq largely implicate CRCs as mediators of drug-induced changes to the chromatin landscapes. Walker et al. (2019) identified wide-ranging cocaine-associated changes in ISWI subunit expression, including changes to Arid3a, -4a, -5a, and -5b and altered expression of Smarca1 and Smarca5 [134]. In addition, chronic cocaine exposure is associated with decreases in BAZ1A expression in human subjects and rodents [135]. Moreover, Arid4a expression is increased in the striatum of patients who had a history of chronic heroin use [136]. Lastly, chromatin remodeling was identified in the NAc following repeated cocaine administration, which suggests that previously identified changes in Baz1a expression may mediate cocaine-induced nucleosome remodeling. Interestingly, BAZ1A contributes dissociable functions within NAc-mediated cocaine-associated behavior, as BAZ1A overexpression in the NAc enhanced cocaine-induced locomotor sensitization, but attenuated acquisition and expression of cocaine-induced CPP.

The ISWI family of CRCs are enticing targets for future SUDs research. BAZ1A contains PHD- and bromodomains, which recognize methylated and acetylated histone, respectively. Perhaps, loss of BAZ1A from ISWI CSCs alters chromatin targeting, leading to re-organization to gene accessibility. However, the mechanism by which BAZ1A contributes to drug-induced chromatin remodeling has yet to be mechanistically demonstrated.

As nBAF contains several neuron-specific subunits (CREST, BAF53b, BAF45b/c), it is the ideal target to investigate CRC’s role in drug-induced changes in gene expression and neuron function. Several studies have identified changes in BAF-specific subunit expression throughout circuitry regulating reward in response to various drugs of abuse. For example, forced abstinence from chronic cocaine increases *Baf190* expression in the striatum [137]. This increased expression coincided with increased binding to SMAD3, which has previously been linked with regulating genes necessary for neuronal plasticity. Additionally, several other BAF-specific subunits have been identified in cocaine-induced dysregulation. The BAF53b subunit is induced by acute cocaine in the accumbens but is downregulated following forced abstinence from chronic cocaine in the ventral hippocampus [138,139,140]. Recent sequencing studies have also identified wide-ranging changes in BAF-specific subunits in response to various drug treatments. Walker et al. (2019) identified changes in response to acute and cocaine re-exposure to *Baf60a, Baf60b,* and *Baf170* in addition to withdrawal-induced changes to *Baf60a* and *Baf47* [134]. Interestingly, acute cocaine downregulated BAF53a in the hippocampus, implicating changes in glial CRC function, as BAF53a is expression is explicitly non-neuronal.

Although most CRC studies focus on cocaine, other drugs of abuse are known to induce changes in BAF-associated gene expression. Post-mortem striatal tissue from chronic heroin users contained elevated levels of *Smarca1* and decreased levels of *Baf60a* and *Baf57* [136]. Alcohol has also been demonstrated to establish long-lasting changes in BAF CRC function. Recently Mathies et al. (2017) identified several SNP clusters within BAF-genes that correlate with alcohol use disorder in humans, including *Baf47*, *Baf60a*, and *Crest* [141]. Lastly, ethanol exposure during neuronal development induces expression of *Baf53b*, *Baf57*, *Baf60*, *Baf200*, and *Crest* [142]. While these studies demonstrate that drugs of abuse are capable of altering nBAF, they fail to establish a mechanistic link between nBAF-mediated CRC function and long-lasting drug-associated behavior.

Research that combined sequencing studies, to identify dysregulated BAF-target genes, with targeted manipulations has highlighted potential mechanisms by which CRCs regulate drug-associated memory and behavior. Loss of function mutations to BAF53b (either through domain-specific or gene-wide deletion) have been shown to produce impairments in LTP in the nucleus accumbens and deficits in cocaine-associated memory formation [81]. These deficits in cocaine-associated memory and synaptic plasticity are both rescued by overexpression of BDNF into the nucleus accumbens. As is the case with BAF53b experiments that utilize hippocampus-dependent memory tasks, the rescue experiments targeting BAF53b in the nucleus accumbens illustrate that the deficits in cocaine-associated memory is not permanent and might not be based solely on abnormal neurodevelopment. Similarly, CREST deletion in the nucleus accumbens blocks LTP induction, cocaine-associated memory formation, and cocaine self-administration [143]. Lastly, BRG1 function is regulated by cocaine, as cocaine abstinence alters BRG1 (and presumptive nBAF complex) binding to target genes, including *Ctnnb1*, *Mef2d*, and *Dbn1*. Subsequent overexpression of *Brg1* in the nucleus accumbens led to enhanced cue-primed reinstatement of cocaine-seeking behavior [144]. These studies suggest that drugs of abuse induce long-lasting changes in circuit plasticity and drug-seeking behavior through dysregulation of BAF-associated CRC function.

## 7. Discussion

Learning events that result in long-term memory formation are known to engage the transcriptional machinery. Recently, the field of neuroscience has acquired a better understanding of how changes to chromatin structure during learning enable stable changes in gene expression, cell function, and memory formation. As previously discussed, epigenetic mechanisms play a substantial role in regulating the transcriptional landscape to promote or hinder memory-related gene expression. Pharmacological and genetic approaches that disrupt histone-modifying enzymes’ function result in aberrant gene expression and synaptic plasticity. Furthermore, the strength and persistence of various types of memories have been shown to depend on the activity of these same epigenetic enzymes. Histone modifications do not occur in isolation, as H3S10 histone phosphorylation has been shown to promote histone acetylation at multiple lysine residues [45]. Recent work has also demonstrated that histone modifications can interact with CRCs to affect learning and memory. Specifically, the CREST subunit of the nBAF complex has been demonstrated to directly interact with CBP, a histone acetyltransferase critical for CREB-targeted gene expression [75]. Moreover, loss of HDAC3 function in the dorsal hippocampus restores memory deficits seen in BAF53b mutant mice [145]. There is an open question as to the extent of the interplay between these epigenetic mechanisms during memory processes. However, researchers should continue to use insights gained from histone modification experiments to guide studies aimed at understanding the ways that long-term memories are primed, encoded, and maintained through under-examined epigenetic mechanisms, such as chromatin remodeling.

New evidence has implicated chromatin remodelers from the ISWI and BAF families in regulating transcriptional processes underlying various forms of cognition. Enzymes from these families contain recognition domains that interact with other epigenetic marks in the developing brain [84]. While in the adult brain, CRCs from these families are selectively recruited in various forms of psychiatric disorders, including SUD and MDD. While critical functions of the nBAF complex, in particular, have been elucidated, much remains unknown about its role in memory and cognitive disorders. Although sequencing studies have identified presumptive gene targets of the nBAF complex, it remains unclear if nBAF has widespread targeting throughout the adult neuronal genome or if nBAF shares gene targets with other CRC families (such as NuRD or ISWI). Furthermore, there is the open question of which nBAF subunits contribute to gene-specific targeting by the complex.

As a neuron-specific subunit of the nBAF complex, BAF53b appeared to be a candidate subunit for targeting nBAF to gene loci. However, loss of BAF53b disrupts but does not abolish nBAF-targeted gene expression [73]. It is possible that nBAF also targets genes through its other neuron-specific subunits, BAF45b/c, but that remains unclear. Nevertheless, a more thorough understanding of the function provided by each of the nBAF subunits in needed. Another avenue for future research is elucidating the mechanism by which learning events establish large-scale changes to chromatin structure. Chromatin remodeling regulates chromatin structure, both at the gene-specific level and the level of hetero- and euchromatin. For example, in addition to active genes being less compacted, highly expressed genes have been shown to change their location in the nucleus [146,147,148], a process dependent on normal CRC function. Drugs of abuse can cause wide-spread changes in gene accessibility [133,136], similar to those seen following memory formation. Next-generation chromosome conformation capture (3C) technologies should be used in conjunction with targeted CRC manipulations to assess how CRCs may regulate various levels of chromatin compaction and orientation in the nucleus during maladaptive memory formation. Given the comorbidity of SUD and MDD, understanding the role of individual CRCs and their subunits in memory processes may provide therapeutic targets for the treatment of both psychiatric disorders.

## Figures and Tables

**Table 1:** Histone modifying enzymes regulate long-term memory.

Histone Modifying Enzyme Class	Member	Target Residues	Effect on Long-Term Memory	References
Acetyltransferase	KAT2A (GCN5)/KAT2B (PCAF) CBP (KAT3A)/p300 (KAT3B)	**H3**: K9, K14, K18, K23 **H4**: K8, K12 **H2A**:K5; **H2B**: K12, K15; **H3**: K14; **H4**: K5, K8	Permissive Permissive	[2,29,31,48,49,50,51,52,53,54,55]
Histone Deacetylase	HDAC1	**H2A**: All; **H2B**: All; **H3**: All; **H4**: All	Repressive	[2,50,51,56,57,58]
	HDAC2	**H2A**: All; **H2B**: All; **H3**: All; **H4**: All		
	HDAC3	**H2A**: All; **H2B**: All; **H3**: All; **H4**: All		
	HDAC4	**Enzymatically Non-Deacetylating**		
	HDAC5	**Enzymatically Non-Deacetylating**		
	HDAC6	**H4**: K5, K8		
Lysine Methyltransferase	KMT1C (G9a)	**H3**: K9	Both	[2,33,34,50,51,59,60,61,62]
	KMT1D (GLP)	**H3**: K9	Both	
	KMT2A (MII1)	**H3**: K4	Permissive	
	KMT2B (MII2)	**H3**: K4	Repressive	
	KMT6A (EZH2)	**H3**: K27	Repressive	
Lysine Demethylase	KDM1	**H3**: K4, K9; **H4**: K20	Permissive	[2,37,50,51,59]
	KDM4B	**H3**: K4 K9	Both	
	KDM5C	**H3**: K9	Both	
	KDM6A	**H3**: K27	Repressive	
